# Post-Sandy Preparedness Policies Lag as Sea Levels Rise

**DOI:** 10.1289/ehp.1307095

**Published:** 2013-07-01

**Authors:** Kim Knowlton, Miriam Rotkin-Ellman, Perry Sheffield

**Affiliations:** 1 Health and Environment Program, Natural Resources Defense Council, New York, New York, E-mail: kknowlton@nrdc.org; 2 Health and Environment Program, Natural Resources Defense Council, San Francisco, California; 3 Department of Preventive Medicine, Icahn School of Medicine at Mount Sinai, New York, New York

In “The Long Road to Recovery: Environmental Health Impacts of Hurricane Sandy” in the May 2013 issue of *Environmental Health Perspectives,* Manuel (2013) provided critical insight from the frontlines about ongoing environmental health threats from Hurricane Sandy. While health risks rise along with the sea levels, policies to help prepare coastal communities for future threats are lagging. Without new policies that reflect the lessons of Hurricane Sandy and a changing climate, this opportunity to improve preparedness will be lost and the health of coastal residents will continue to be threatened.

The storm surge accompanying Hurricane Sandy caused an all-time record-breaking flood height of 13.88 ft, which included effects of an estimated 9.23-ft flood surge (Freedman 2012). Before Sandy, flood projections based on global climate change did not include this level of storm surge until the 2050s, when it was expected to result from a 100-year storm event (New York City Panel on Climate Change 2009). We don’t have as much time to prepare as we thought we did. The tools we use to plan for storm flooding, especially floodplain maps, need to be quickly updated to fully reflect the new realities of climate change. Many parts of New York City that were inundated during Sandy were not within Federal Emergency Management Administration (FEMA) flood zones and thus were not prepared for flooding. Half of all the affected residences and half of affected buildings of all types were outside of FEMA’s then-mapped 100-year floodplain (New York City Special Initiative for Rebuilding and Resiliency 2013).

Policy gaps are hampering preparations for the storms that we can expect with climate  change. In early 2013, FEMA updated the flood risk maps for much of the New York–New Jersey region, some of which had not been updated since 1983 (FEMA 2013). The new flood maps of New York City and Westchester County showed 35,000 more structures at risk of flooding, doubling the numbers of at-risk structures (Buckley 2013). Unfortunately, the updated maps do not include future vulnerability from climate change (Bagley 2013). FEMA has a policy prioritizing agency-wide integration of climate change adaptation in planning and actions (FEMA 2012), but it failed to move forward on directives to evaluate climate change implications on disaster planning and on the National Flood Insurance Program. Now in the aftermath of Sandy, FEMA must take action. The agency can make a difference in coastal communities by requiring states to account for climate change in their hazard mitigation planning. The Biggert-Waters Flood Insurance Reform Act, signed into law in July 2012, further aims to improve floodplain maps (National Association of Realtors 2013). However, FEMA has not yet committed to a timeline for producing new maps that incorporate the latest climate vulnerability projections, and in the meantime, communities are at risk from rising sea levels and storm surges influenced by climate change.

Hurricane Sandy is a stark example of the cost of inaction and not being prepared. Recent estimates are that Sandy cost at least $70 billion in damages, with only half of that insured (SwissRe 2013). Research shows that the short-term damages are the tip of the iceberg. For example, long-term mental health distress and disability were pervasive post-storm effects among people displaced after Hurricane Katrina, > 2 years after the storm (Abramson et al. 2008). Factoring in the health impacts from hurricanes can raise cost estimates by hundreds of millions of dollars—billions when mortality valuation is included (Knowlton et al. 2011). Preparedness for flood events has been shown to be cost effective, with benefits of efforts to reduce losses from natural hazards outweighing the costs by a factor of 5 to 1 for flooding (Rose et al. 2007).

Hurricane Sandy was a wake-up call not limited to the Northeast: An estimated 53% of Americans live in coastal counties now, and that is excpected to reach 75% by 2025 (National Institute of Environmental Health Sciences 2009). Because of climate change, hurricane storm surges are worse than in the past, and hundreds of millions of people must be prepared. The public health concerns highlighted by Manuel (2013) must be translated into actionable, up-to-date preparedness policies at the federal, state, and local levels. Climate change is a matter of health, fueling extreme weather events that can challenge even strong, resilient people. By taking the climate-preparedness challenge seriously, we stand to save lives, save dollars, and create healthier, more secure communities. Let’s not squander Sandy’s call to get ready while we still can.
